# Cumulative Loss of Inner Retinal Neurons in Healthy and Glaucoma Subjects

**DOI:** 10.1167/iovs.67.11.10

**Published:** 2026-09-04

**Authors:** Kazuhiro Kurokawa, James A. Crowell, Yan Liu, Marcel Bernucci, William H. Swanson, Donald T. Miller

**Affiliations:** 1 School of Optometry, Indiana University, Bloomington, Indiana, United States

**Keywords:** adaptive optics (AO), retinal ganglion cells, optical coherence tomography (OCT), glaucoma, high-resolution retinal imaging

## Abstract

**Purpose:**

Considerable ganglion cell (GC) loss occurs over the normal human lifespan and even more in ocular diseases such as glaucoma. Current clinical methods, however, lack sufficient resolution and sensitivity to directly detect GC loss. Here, we used adaptive optics optical coherence tomography (AO-OCT) to overcome these limitations, enabling longitudinal tracking of individual GC somas and direct measurement of their loss in healthy and glaucomatous eyes.

**Methods:**

Six healthy subjects and two patients with glaucoma were imaged at multiple retinal locations using AO-OCT. Healthy subjects were imaged annually for up to 3 years (4 total timepoints); patients with glaucoma were reimaged 2 to 3 years after baseline. Individual ganglion cell layer (GCL) somas were identified and tracked across sessions and soma densities were quantified. A flicker-based image comparison approach enabled sensitive detection of soma loss from which cumulative loss was determined by subject and location.

**Results:**

The GCL soma mosaic was highly stable, with 23,586 somas tracked in healthy subjects. Soma densities agreed with histologic values, including near the fovea, and correlated strongly with GCL thickness (*R*^2^ = 0.96). The mean soma loss rate in healthy subjects was −0.10%/year (95% confidence interval [CI] = −0.09 to −0.11%/year), with no significant differences across subjects or locations. By contrast, one glaucoma patient showed a loss rate within an arcuate defect over 100× greater (−31.6%/year; corresponding GCL thinning rate of −13%/year), whereas the other demonstrated a near-normal rate (−0.26%/year).

**Conclusions:**

In vivo measurement of individual GC soma loss is now feasible and provides a sensitive tool for longitudinal evaluation of GC health.

The human retina contains approximately one million ganglion cells (GCs; range = 0.7 to 1.5 million),^1^ a population believed to decline gradually throughout the normal human lifespan in the absence of pathology.^2^ Thus, aging is detrimental to GCs and ultimately to vision. Given their high metabolic demand, GCs are thought to be especially vulnerable to age-related ocular changes; key among these are reduced retinal blood flow, stiffening of the lamina cribrosa at the optic nerve head, and diminished mitochondrial energy production.^3^ Together with secondary influences from genetic and environmental factors, these changes are thought to determine an individual's rate of GC loss.

Because these changes increase the susceptibility of GCs to injury, they have also been implicated in various neurodegenerative conditions. Aging is a major risk factor for glaucoma,^4^^–^^6^ Parkinson's disease,^7^^–^^9^ Alzheimer's disease,^8^^–^^10^ and amyotrophic lateral sclerosis.^8^^,^^11^ Given these associations, it has been postulated that delaying or even reversing these age-related changes could improve GC health and ward off disease. Although this notion may suggest possible therapies targeting the responses of GCs to aging, informed treatment requires timely and precise measurements of an individual patient's rate of GC loss in order to develop an optimal personalized intervention strategy.

Age-related loss of both GC somas and optic nerve axons has been extensively studied histologically, with the number of both consistently declining with age. Linear regression estimates of this decline (expressed as the slope normalized to the intercept) indicate exceedingly small annual loss rates with considerable variability across studies. Reported rates range from −0.19% to −0.57% per year for somas^12^^–^^15^ and from −0.07% to −0.4% per year for axons.^16^^–^^21^ Importantly, many of these estimates are not significantly different from zero, highlighting the inherent difficulty of quantifying age-related GC loss using cross-sectional histologic approaches. The combination of substantial inter-subject variability and shallow rates of decline limits the ability of such approaches to detect small but biologically meaningful changes over time.

Direct measurement of GC somas in the living human eye would allow longitudinal tracking of soma loss in the same eyes, thereby eliminating inter-subject variability. However, in vivo assessment of GC populations remains exceedingly challenging. To address this difficulty, clinical studies have relied on surrogate measures of visual sensitivity and thicknesses of inner retinal layers from which GC number and loss in aging and disease are inferred. Early work compared loss of visual sensitivity to GC counts derived from histological data in humans^22^^,^^23^ and macaque monkeys.^24^^–^^26^ These foundational studies established the first structural-functional relationships between visual performance and GC counts. More recent methods estimate GC axon counts from optical coherence tomography (OCT) measurements of retinal nerve fiber layer (RNFL) thickness around the optic nerve,^26^ although such estimates require assumptions about non-neural tissue contributions that OCT cannot directly resolve.^27^^,^^28^ Today, an extensive collection of structure-function models exist that attempt to infer GC population from clinical measurements of structure and function,^29^^–^^34^ which now include thickness measurements of the ganglion cell layer (GCL) and inner plexiform layer (IPL).^35^ These methods have made substantial progress, but as intersubject variability frequently results in disagreement between the structural and functional measures, the clinical usefulness of structural-functional comparisons remains challenging.^36^^,^^37^

A fundamental limitation of these clinical methods is their reliance on indirect GC-count surrogates calibrated to population-averaged histological data. This limitation is compounded by the extremely low rate of age-related GC soma loss, which places a premium on measurement sensitivity. Sensitivity must reach the level of individual GCs to detect GC loss as it occurs.

Here, we address these challenges using recent advances in high-resolution retinal imaging based on adaptive optics OCT (AO-OCT), which enables direct visualization and quantification of individual GCL somas in both healthy subjects^38^^–^^44^ and patients with glaucoma.^44^^–^^47^ In this study, we tested the hypothesis that AO-OCT provides sufficient resolution and sensitivity to longitudinally image and track the loss of individual GCL somas, by repeatedly imaging six healthy subjects and two patients with glaucoma over several years. We show that AO-OCT can quantify GCL soma loss rates associated with normal aging and glaucomatous progression, revealing notably different rates between the two. We compare these measured rates with previously reported histological and clinically inferred estimates. To the best of our knowledge, this study provides the first longitudinal measurements of individual GCL soma loss in the living human retina. Parts of this work were previously reported in a conference proceeding^48^ and in abstract form^49^^–^^52^ (K. Kurokawa, ARVO Abstract #1781, 2019; K. Kurokawa, ARVO Abstract #201, 2020; and H. W. Jung, ARVO Abstract #229, 2020).

## Methods

### Subjects

Six subjects (34.2 ± 11.4 years old at baseline, mean ± SD) with no history of ocular disease and two patients diagnosed with glaucoma (61 and 71 years old at baseline) participated in the study (Table 1). The healthy cohort included one subject (H053) who was being treated for ocular hypertension with latanoprost but was otherwise healthy (see Table 1 footnote for H053 characterization; see Discussion for loss rate analysis excluding H053). The two patients (subjects G142 and G147) had open-angle glaucoma with inferior arcuate retinal nerve fiber layer defects as determined by Spectralis OCT images (Heidelberg Engineering, Heidelberg, Germany) and corresponding superior arcuate visual field defect as determined by Humphrey visual fields (Zeiss, Dublin, CA, USA). Glaucoma varied in type and stage of disease, with subject G142 having primary open-angle glaucoma (POAG) and subject G147 having normal tension glaucoma (NTG).

**Table 1.** Subject Information

Subject ID	Eye	Health State	Age^†^, Y	Sex	Eye Length, mm	IOP, mm Hg	Imaging Protocol, See Table 2	Timepoints^‡^
H127	OD	Healthy	24–27	M	23.98	16–19	A	Baseline (T0), 1 y (T1), 2 y (T2), 3 y (T3)
H116	OD	Healthy	26–29	M	24.04	10	A	Baseline (T0), 1 y (T1), 2 y (T2), 3 y (T3)
H125	OD	Healthy	47–50	F	23.27	11	A	Baseline (T0), 1 y (T1), 2 y (T2), 3 y (T3)
H053	OD	Healthy^*^	50–53	M	25.4	16–21^*^	B	Baseline (T0), 1 y (T1), 2 y (T2), 3 y (T3)
H154	OD	Healthy	27–28	M	25.24	14–16	A	Baseline (T0) and 1 y (T1)
H155	OD	Healthy	31–32	M	24.75	19–21	A	Baseline (T0) and 1 y (T1)
G142	OS	POAG	71–73	M	22.46	13–20	C	Baseline (T0) and 2 y (T2)
G147	OD	NTG	61–63	F	24.56	12–14	C	Baseline (T0) and 2 y (T2)

F, female; M, male; NTG, normal tension glaucoma; POAG, primary open-angle glaucoma; mm Hg, millimeters of mercury.

* Under treatment for ocular hypertension with latanoprost but was otherwise normal, including normal optical nerve head appearance (cup-to-disc ratio of 0.4), 24-2 visual fields, and family history. Has retained normal visual fields and ocular health a decade after baseline AO-OCT measurements were acquired.

† Age at first and last imaging sessions.

‡ T0 to 3 are labels to denote the measurement timepoints.

G142 was diagnosed at 60 years of age, a decade before participating in the present study, and had been treated since with cosopt and travoprost. G142 had VF 24-2 mean deviation (MD) and pattern standard deviation (PSD) of −7.9 decibel (dB) and 12.22 dB, global circumpapillary RNFL thickness (cpRNFLT) of 56 µm, and macular ganglion cell complex thickness (mGCCT) of 66.5 µm at baseline timepoint, all of which fall outside the normal limits (see Supplementary Fig. S1). G147 was diagnosed with glaucoma at 61 years of age, the same time as participating in the present study, and had been treated since with cosopt and latanoprost. G147 had VF 24-2 MD and PSD of −2.8 dB and 6.41 dB, global cpRNFLT of 84 µm and mGCCT of 88.1 µm at baseline timepoint, which collectively indicates abnormality (see Supplementary Fig. S2).

Subject G142 also had glaucomatous damage in the superior hemifield with corresponding inferior visual field defects. Although we could have imaged the superior defect for this study, we selected the inferior one as it was more centrally located and better defined, thus providing a clearer transition from relatively healthy to severely diseased retina.

All subjects received a comprehensive eye examination within 3 years of the study baseline measurements and had best-corrected visual acuity of 20/20 or better, clear optics, and dilated pupil larger than 5 mm. Axial eye length was measured by IOLMaster (Zeiss, Oberkochen, Germany) and used to correct for axial length differences in scaling of the retinal images following the method of Bennett et al.^53^ Both Spectralis OCT and scanning laser ophthalmoscopy (SLO) images were acquired and used to locate the subject's fovea, identify retinal regions to be reimaged, and generate spatial maps of GCL thickness.

We obtained written informed consent after the nature and possible risks of the study were explained to the subjects. All protocols adhered to the tenets of the Helsinki Declaration and were approved by the Institutional Review Board of Indiana University.

### AO-OCT Imaging Procedure

All GCL soma images used in this study were acquired with the Indiana AO-OCT imaging system.^54^^,^^55^ The system was configured in its two-camera mode to achieve an acquisition speed of 500 K A-scans/s. The optical power delivered to the eye was below 430 μW (λ_c_ = 790 nm; Δλ_FWHM_ = 42 nm), which is within the American National Standards Institute (ANSI) safety limit for the imaging protocol used.^56^ Axial and lateral resolutions were 4.7 µm in retinal tissue (*n* = 1.38) and 2.4 µm, respectively. Pixel sampling was approximately 1 µm in all dimensions. System focus was varied in 7 to 11 µm (0.02–0.03 diopter) steps to cover the entire GCL, maximizing signal strength and sharpness of the GCL soma reflections.

One drop of 1% tropicamide was administered to the eye to induce mydriasis and partial cycloplegia prior to the imaging session. A dental impression mounted on a motorized XYZ translation stage helped stabilize the subject's eye and head and align the eye to the AO-OCT system. The subject fixated on an approximately 1-degree cross target displayed on a liquid crystal display, which was moved to different locations to allow imaging of targeted retinal locations with the AO-OCT, as described below.

We acquired AO-OCT volumes (450 × 450 × 512 pixels in XYZ dimensions) of 1.5 degrees × 1.5 degrees retinal patches at the targeted locations (Table 2). At each location, we acquired 10 to 15 videos (12 volumes/video), each 5 seconds in duration, resulting in 34 to 56 GB of raw data. In all six healthy subjects, we imaged five locations temporal to the fovea (1.5–3 degrees, 3–4.5 degrees, 6–7.5 degrees, 8–9.5 degrees, and 12–13.5 degrees), and in one subject, H053, we imaged an additional two locations nasal to the fovea (1.5–3 degrees and 3–4.5 degrees). Except for this subject, all imaging was completed within a 2-hour session, including pupil dilation, subject alignment to the AO-OCT, rest breaks, and data transfer. At some eccentricities, the vertical (superior-inferior) positioning was slightly adjusted to include retinal vasculature, which helped confirm the location in subsequent imaging sessions.

**Table 2.** Imaging Protocols and Retinal Locations Imaged

Protocols	*N* of Retinal Patches	Retinal Eccentricity	Subject ID
A	5	1.5–3°T, 3–4.5°T, 6–7.5°T, 8–9.5°T, 12–13.5°T	H127, H116, H125, H154, H155
B	7	Protocol A plus 1.5–3°N and 3–4.5°N	H053
C	3 to 4 adjacent	4–7°NI	G142, G147

T, temporal to the fovea; N, nasal to the fovea; NI, nasal-inferior to the fovea.

In the glaucoma patients, we acquired volumes at three to four adjacent locations that traversed into the arcuate defects, the edges of which were determined using GCL thickness and attenuation coefficient maps. The initial imaging location was positioned 3 degrees to 4 degrees nasal and 2 degrees to 6 degrees inferior to the fovea, depending on the eye, which corresponded to a radial eccentricity of 4 degrees to 7 degrees. All images were acquired within a 2-hour session.

After data collection, we registered and averaged volumes (typically 150–200 per retinal location) to improve the signal-to-noise ratio and image contrast of the somas.^38^^,^^42^ Processing required several hours per retinal location. We then manually identified individual GCL somas (discussed below in Data Analysis) and quantified their count and density. Additionally, we measured the local GCL thickness from each average volume. To do this, we projected the AO-OCT volume along its vertical dimension and manually segmented the inner and outer GCL boundaries, corresponding to the interfaces with the NFL and IPL, respectively. We then applied linear fits to the segmented boundaries and averaged to estimate local GCL thickness.

To longitudinally track individual somas, we acquired AO-OCT volumes of the same retinal patches in the first (baseline, T0) session and at 1 year (T1), 2 years (T2), and 3 years (T3) later for the healthy subjects, and at 2 years later for the patients with glaucoma (see Table 1, rightmost column). We controlled the location of the imaged retinal patch by varying the fixation position and were able to return to the same location years later by comparing real-time en face projections to images from the baseline session. Blood vessels in the Spectralis SLO images served as a coarse guide, and we fine-tuned the location using the pattern of blood vessels and nerve fiber bundles displayed in real-time in en face view by our AO-OCT control software. After collecting 150 to 200 volumes for a given retinal patch, we registered them to each other and averaged the registered volumes. We did the same registration and averaging for each subsequent yearly session. The average registered volume from the baseline session was then used as a reference for registering all of the yearly sessions back to the baseline. These average volumes had sufficient image quality and overlap with each other to allow identification and tracking of GCL somas across the imaging sessions.^40^ For the four retinal eccentricities closest to the fovea (1.5–3 degrees, 3–4.5 degrees, 6–7.5 degrees, and 8–9.5 degrees) in the healthy subjects, we selected a fixed subregion of interest (0.67 degrees field of view [FOV]) from the area common to all measurements (T1, T2, and T3) for further analysis. At the 12 degrees to 13.5 degrees location, a 4× larger sub-region (approximately 1.3 degrees FOV on average) was used to offset the smaller number of somas at this retinal eccentricity. For the glaucoma patients, we analyzed a single subregion within the AO-OCT composite image. The subregion located within the arcuate defect and subtended 1.4 degrees × 1.4 degrees for G142 and 1 degree × 1 degree for G147.

### Data Analysis

We determined the annual soma loss rate for each subregion of interest in each subject. The loss rate was defined as the ratio of somas lost over a 1-year period to the total somas counted at baseline and was expressed as percent per year. Soma counting was performed by trained human graders, except for T3 in subjects H127, H116, H125, and H053, and for T0 and T1 in subjects H154 and H155 for which machine-learning software^46^ was used instead to expedite the analysis, although manual checking was still required. The software was previously developed using a weakly supervised deep learning algorithm for automated, high-throughput soma identification in which a subset of our AO-OCT images and manual annotations was used to train a convolutional neural network and validate its predictions.

To minimize systematic error, we implemented the following multi-step, multi-grader procedure that first determined soma counts and densities and then soma loss. Because soma loss is very small, its measurement required substantially higher accuracy than could be achieved with either manual or machine-learning counting alone. Additional steps, namely reconciliation of counts and comparison of flickering images, were therefore required to achieve the sensitivity needed to determine cumulative loss by subject and retinal location (see Supplementary Fig. S3 for the procedural flowchart).

(1) Determine total GCL somas present within sub-region (*N*): Somas were manually identified and counted by trained graders. In Supplementary Table S1, the grader roles for years T0 to T3 are denoted by grader 1 to 4 and the individual agents (human or software^46^) assigned to these roles are denoted by A1 to A8 and AS (for Software^46^). We avoided assigning any individual agent to process data from the same location across multiple sessions in the same subject to minimize effects of systematic bias on the part of any individual agent. This task was facilitated using the customized graphic user interface display window depicted in Supplementary Video S1. En face (XY) and cross-sectional (XZ and YZ) slices display the cursor position, which is moved in real-time by the operator to the volumetric center of each soma for marking. On average, graders counted approximately 100 somas per hour, although the counting rate varied substantially depending on the structural complexity of the GCL.
(2) Reconcile/correct soma counts and determine total GCL somas lost in subregion (L): This task was performed by a subset of more experienced agents, with grader 5 responsible for determining the number of somas lost between years T0 and T1 (Y0–1), grader 6 between years T0 and T2 (Y0–2), and grader 7 between years T0 and T3 (Y0–3). Images of the same retinal patch from the 2 years were displayed on a computer screen in rapid alternation (see Supplementary Video S2). This transformed the difficult task of identifying and counting somas into the much less error-prone task of detecting and classifying differences between two images. For example, soma loss with concomitant inward migration of neighboring cells would manifest as a pattern of alternating inward and outward motion as the grader flipped between images. First, the more experienced grader (e.g. grader 5) would use this task to examine locations of disagreement between the two primary graders for the years in question (e.g. grader 1 and grader 2) and to determine whether the disagreement represented an error on the part of one or both graders or a genuine instance of cell loss. The grader would then examine areas of change between the two images that were not identified as containing GCL somas by the earlier graders in order to see if they had both missed any cells. On average, graders required several hours to process each image pair, although processing time varied substantially depending on the structural complexity of the GCL. We will consider this process in more detail in Discussions, Counting performance.
(3) Determine the annual soma loss rate (%/yr): We performed a regression to fit a line with intercept zero to the cumulative GCL soma losses (expressed as percentages, 100*L/N) against the session times expressed in years (rounded to the nearest day) to account for variation in times between sessions. The fitted slope *a* is our estimate of the annual soma loss rate (%/yr); loss rates were separately estimated for each location, for each subject, and for all of the subjects pooled.

To better characterize performance of our soma counting and loss detection methods, we analyzed repeated measures of GCL soma density and loss in a subset of AO-OCT volumes. Three graders independently measured density in 10 volumes of the same GCL soma patch in each of 2 subjects and measured soma loss by comparing volumes of the same GCL soma patch acquired 1 year apart.

### Statistical Analysis

Linear regression was used to estimate the rate of soma loss at each retinal location for each subject and for the pooled cohort. A 2-way ANOVA was performed to assess differences in loss rate variance across subjects and retinal locations. A *t*-test was used to compare RGC density measurements obtained with AO-OCT and histology.

## Results

### Marking and Tracking GCL Somas in Healthy Subjects

The trained graders manually marked and tracked 16,607 GCL somas annually over 3 years at 5 to 7 retinal eccentricities (5 temporal and 2 nasal) in 4 of the healthy eyes (H127, H116, H125, and H053). An additional 6979 GCL somas were manually marked and tracked over 1 year at the same 5 temporal retinal eccentricities in the other 2 healthy subjects (H154 and H155). In total, 22 retinal patches were analyzed across 4 timepoints, along with an additional 10 retinal patches analyzed across 2 timepoints.

**Figure 1.**
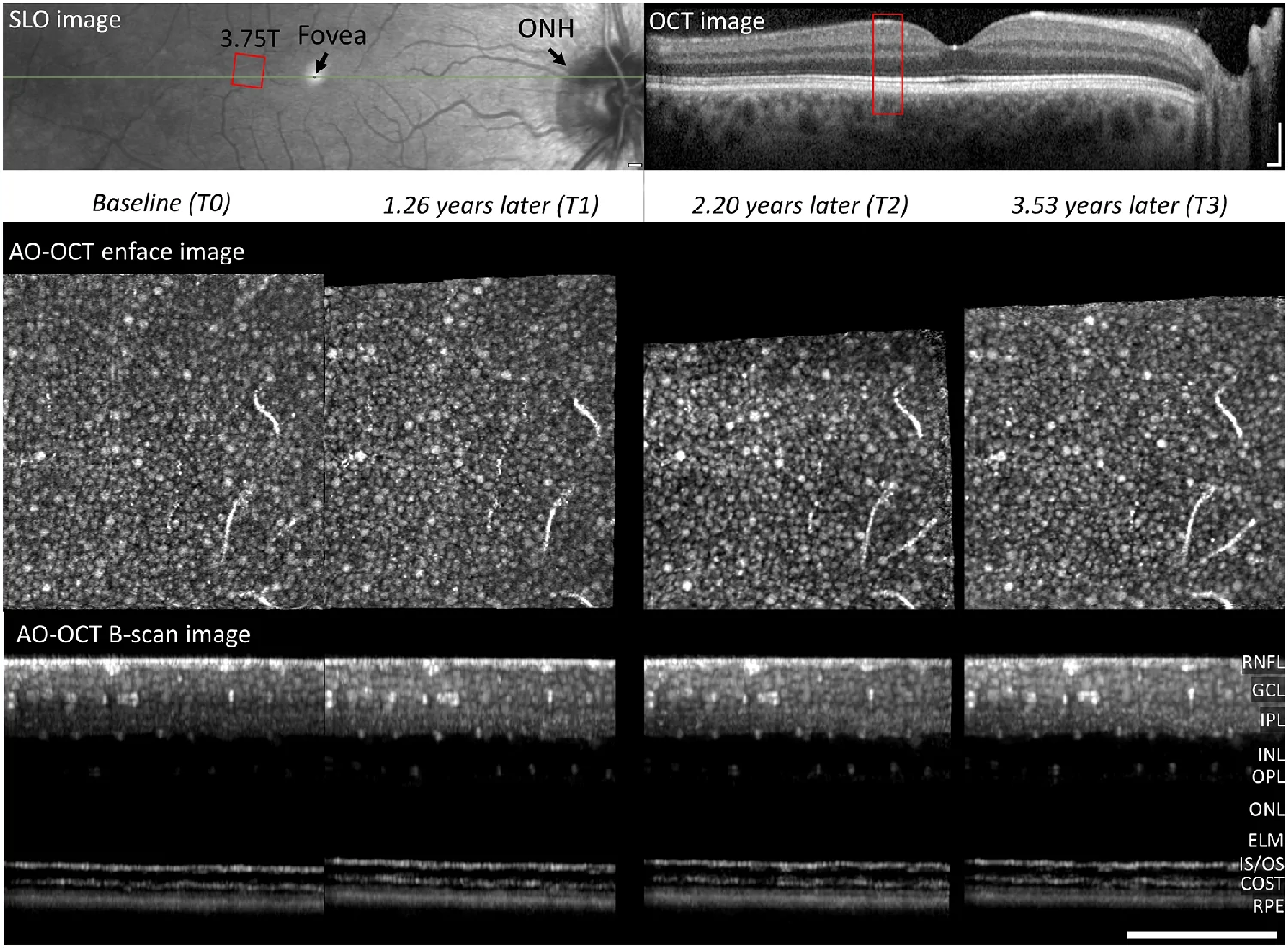
En face and cross-sectional retinal images from subject H053. (*Top row*) Spectralis SLO and OCT images were acquired at baseline. *Red boxes* indicate the 3.75 degrees temporal retinal location (corresponding to 3–4.5 degrees eccentricity) where AO-OCT volume images were acquired. (*Bottom row*) AO-OCT en face and B-scan images were acquired at baseline (T0), 1.26 years (T1), 2.20 years (T2), and 3.53 years (T3) follow-up. System focus was set at the GCL. White *scale bars* indicate 200 µm.

Figure 1 illustrates a representative location tracked longitudinally in subject H053 over 3 years (see Supplementary Fig. S4 for the other 6 locations imaged in this subject). Visual inspection of the AO-OCT images revealed that the GCL soma mosaic was remarkably stable over time, both in the spatial arrangement and reflectance of individual somas. This stability was consistently observed across all healthy subjects (see Supplementary Figs. S4–S9 and Supplementary Videos S3–S8).

**Figure 2.**
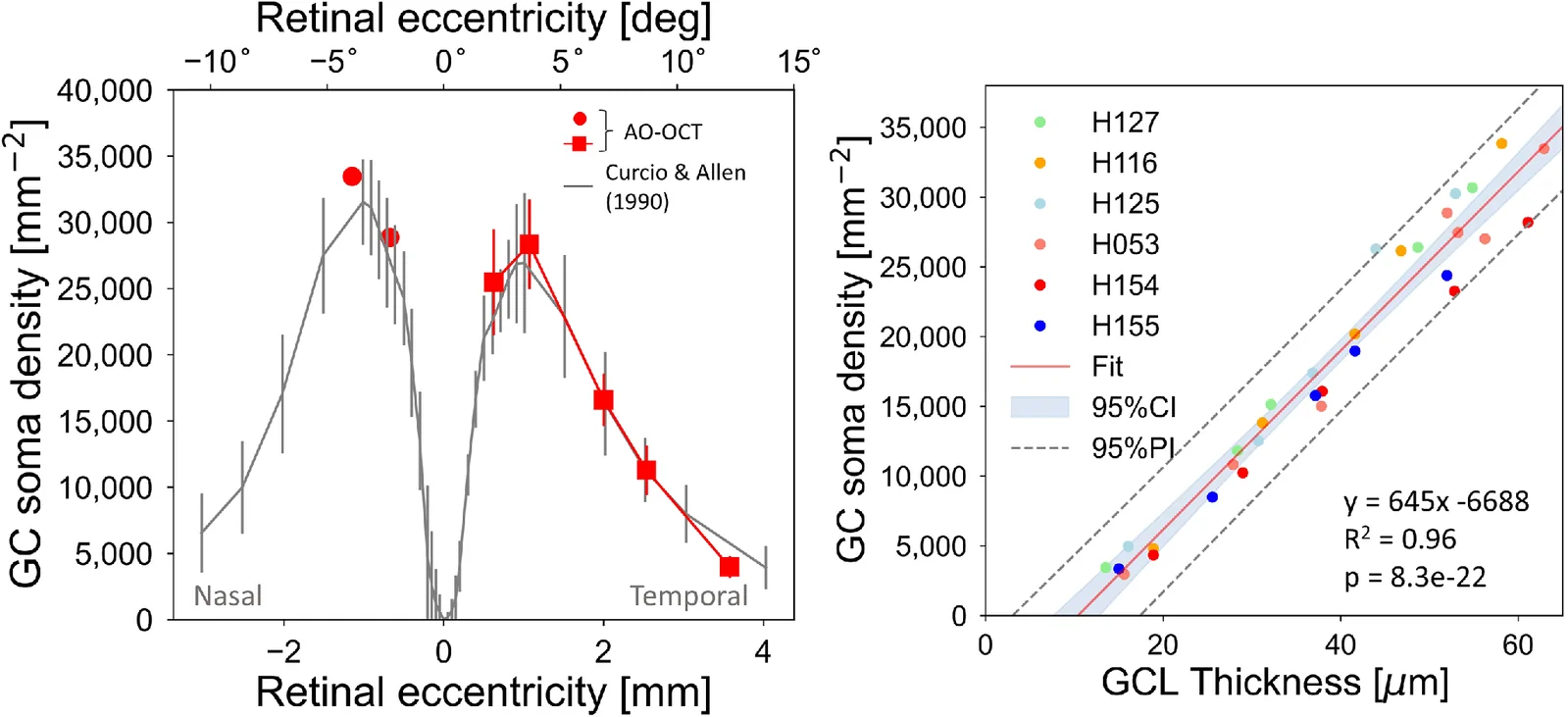
In vivo measurements of GC soma density in healthy subjects. (*Left*) Comparison of GC soma density measured in vivo using AO-OCT with histologic density values reported in the literature.^1^ *Red squares* and *error bars* indicate the mean and SD across the six healthy subjects. The two *red circles* denote GC density measurements obtained nasal to the fovea in subject H053. Note that AO-OCT density measurements are plotted against mean radial retinal eccentricity across the six subjects (see Supplementary Table S2), whereas histologic data are plotted against horizontal retinal eccentricity. For comparison, the upper abscissa is scaled in degrees by converting the lower abscissa (millimeters) using the mean axial eye length of the subjects (24.2 mm).^53^ (*Right*) GC soma density is plotted as a function of GCL thickness measured by AO-OCT for the 32 retinal patches across the 6 healthy subjects. CI, confidence interval; PI, prediction interval.

GC soma density measurements from the 7 retinal locations (5 temporal and 2 nasal) are plotted in red in Figure 2 (left), pooled across all healthy subjects, and listed by subject and retinal location in Supplementary Table S2. Because some images were intentionally offset slightly in the superior-inferior direction to include retinal vasculature for confirming image location, our measured densities are plotted against the mean radial retinal eccentricity calculated from the actual imaging locations for each subject (see Supplementary Table S2). Soma density peaked at about 30,000 somas/mm^2^ at a mean radial retinal eccentricity of 3.64 degrees (nominally the 3 degrees–4.5 degrees location) and decreased monotonically with increasing retinal eccentricity. Histologic data from Curcio and Allen (1990)^1^ are plotted in gray for comparison. Our mean measured densities at each eccentricity were not significantly different from the histologic values^1^ (*t*-test; *P* > 0.05).

Note that because we could not distinguish GC somas from displaced amacrine cell somas in our AO-OCT images, we corrected our density estimates by subtracting the proportion of displaced amacrine cells reported by Curcio and Allen (1990)^1^ from our GCL soma density estimates. This correction followed the approach of Liu et al. (2017).^38^

GC soma density was strongly correlated with AO-OCT measurements of local GCL thickness across all six healthy subjects (see Fig. 2 right; GC density = 645 × GCL thickness – 6628, *R^2^* = 0.96, *P* < 0.001). GCL thickness and soma density ranged from 15 to 60 µm and 3000 to 33,000 somas/mm^2^, respectively. A detailed breakdown of GCL soma density and thickness by retinal location and subject is provided in Supplementary Table S2.

### Measuring GCL Soma Loss in Healthy Subjects

**Figure 3.**
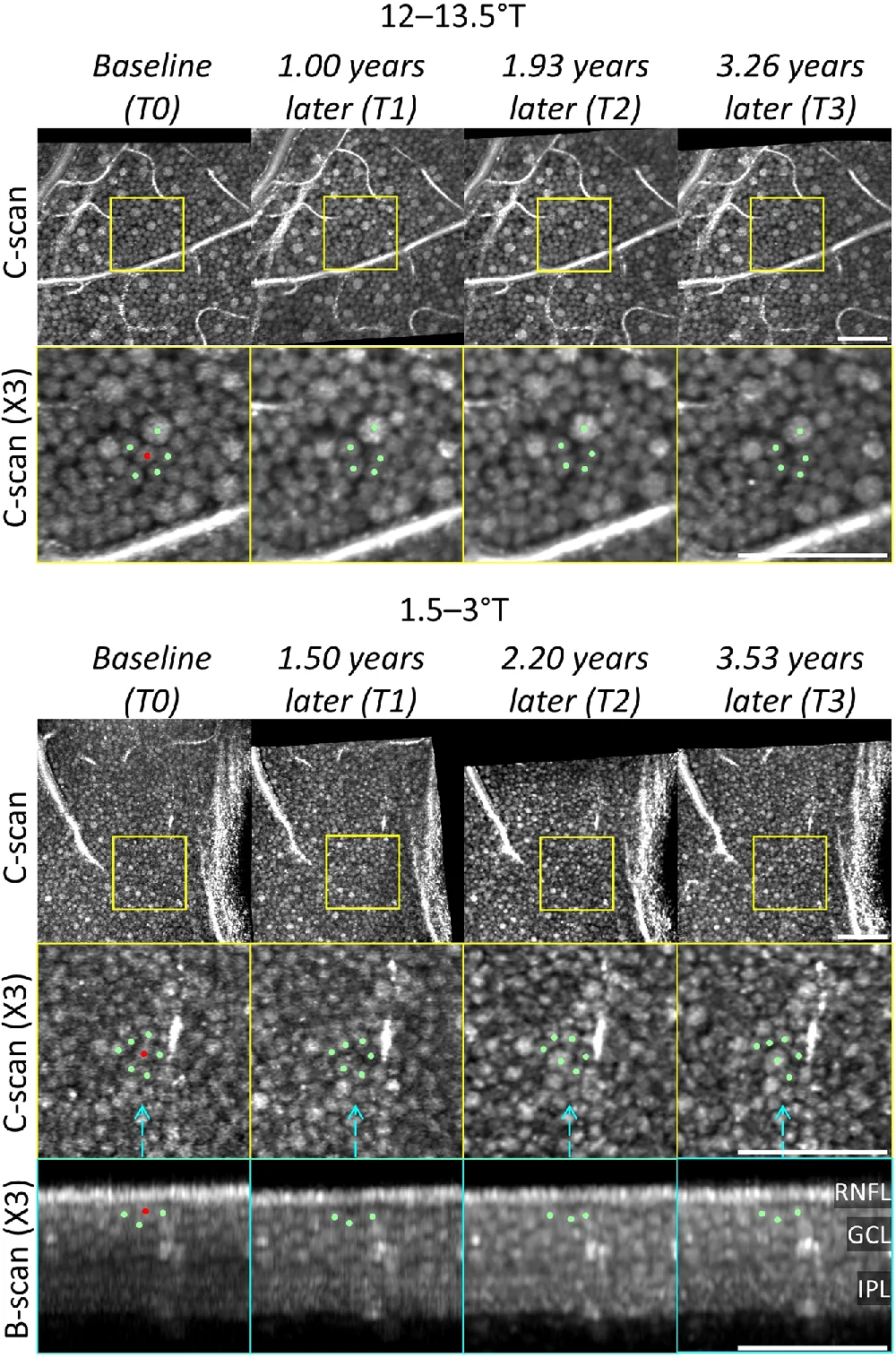
Examples of a GCL soma loss at (*top*) 12 degrees to 13.5 degrees and (*bottom*) 1.5 degrees to 3 degrees retinal eccentricities in subject H053. The lost somas are denoted by *red* markers with surrounding somas denoted by *green* markers. Markers are shown in both en face and sagittal-sectional views. *Blue arrows* denote the B-scan locations on the en face used for these sagittal views. White bars denote 100 µm.

Figure 3 shows representative sequences of AO-OCT images that capture soma losses at two retinal eccentricities in a single subject. As described in the Methods, we used an image alternation technique to facilitate detection of soma loss and displacement events. When viewed in this manner (Supplementary Video S9), soma losses appear as abrupt, localized changes in the GCL mosaic. In the magnified images, red markers point to somas that are lost between the T0 and T1 imaging sessions, whereas green markers denote neighboring somas that migrate into the voids created by these losses.

**Figure 4.**
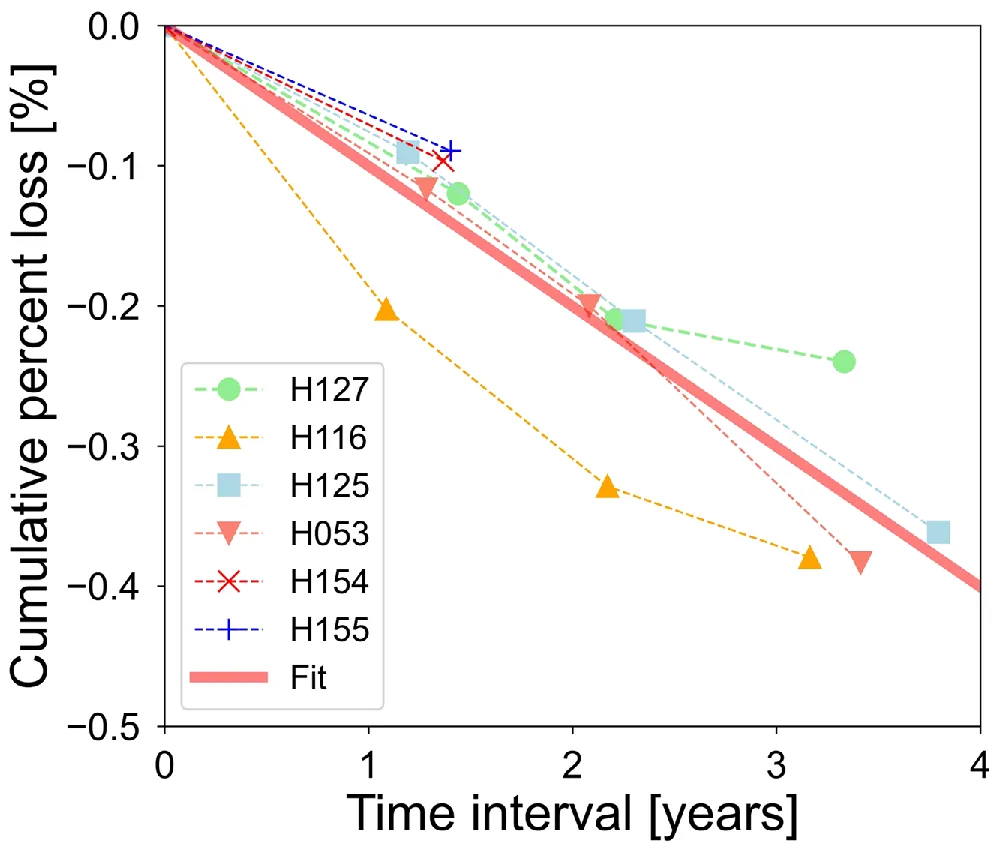
Cumulative percentage loss of GCL somas in the six healthy subjects. Individual data points represent cumulative soma loss as a function of follow-up interval. The *red line* indicates the least-squares linear fit, yielding an overall soma loss rate of −0.10%/yr (*P* < 0.001).

Mean cumulative soma loss across the six healthy subjects (summed over the 5 temporal retinal locations per subject) ± 1 SD was 4.8 ± 1.7, 8.8 ± 2.9 and 12.0 ± 2.9 somas at T1, T2, and T3, respectively (Table 3). The corresponding mean total soma count per subject ± 1 SD was 3562 ± 318. Applying linear regression (as described in Methods) to the measured cumulative percent losses and intersession intervals (rounded to the nearest day) yielded an overall soma loss rate across subjects of −0.10 %/yr (*P* < 0.001; Fig. 4), with a 95% confidence interval (CI) of −0.09 to −0.11%/yr and a prediction interval of +0.02 to −0.22%/yr. A 2-way ANOVA revealed no significant effect of subject on GCL soma loss rate (F(5,20) = 0.37, *P* = 0.86, ω² = 0.00; see Supplementary Table S2) and no significant effect of retinal location, including the two nasal locations imaged in subject H053 (ANOVA; F(6, 20) = 0.85, *P* = 0.55, ω² = 0.00; see Supplementary Table S2).

**Table 3.** GCL Soma Loss in Healthy and Glaucoma Subjects

		Time Interval Starting From T0, Y			Cumulative Loss of GCL Somas Across All Retinal Locations			
Subject ID	Total Tracked GCL Somas	T1	T2	T3	T1	T2	T3	Soma Loss Rate, %/Yr^*^
H127	3,336	1.44	2.21	3.33	4	7	8	−0.080
H116	3,953	1.09	2.17	3.17	8	13	15	−0.134
H125	3,319	1.19	2.30	3.79	3	7	12	−0.093
H053 (temporal^†^)	3,783	1.30	2.14	3.47	5	8	13	−0.099
H154	3,792	1.37	–	–	5	–	–	−0.090
H155	3,187	1.40	–	–	4	–	–	−0.085
Total	21,370	–	–	–	29	35	48	−0.100
Mean ± SD, temporal only	3,562 ± 318	1.30 ± 0.13	2.21 ± 0.07	3.44 ± 0.26	4.8 ± 1.7	8.8 ± 2.9	12.0 ± 2.9	−0.101 ± 0.023
H053 nasal^‡^	2,216	1.23	1.93	3.26	2	4	10	−0.122
G142^§^	167	–	2.30	–	–	1	–	−0.26
G147^§^	136	–	1.72	–	–	74	–	−31.65

* The %/year based on linear regression fit of soma loss.

† The 5 temporal retinal locations.

‡ The 2 nasal retinal locations.

§ Within arcuate defect.

### Measuring GCL Soma Loss of Glaucoma Subjects

**Figure 5.**
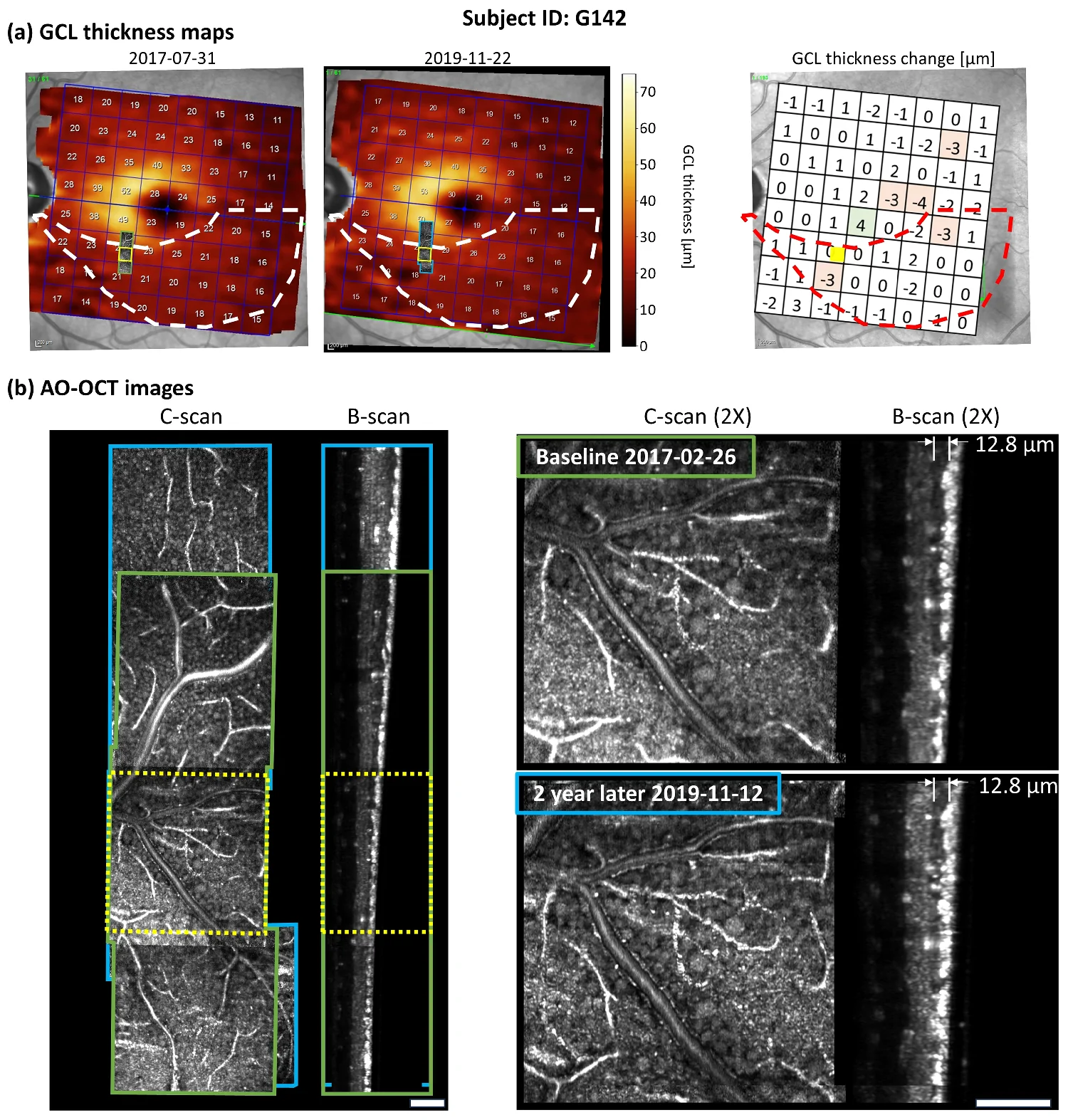
Longitudinal assessment of glaucomatous damage in subject G142. (**a**) GCL thickness maps measured with Spectralis OCT at baseline (*left*) and 2 years later (*middle*). The corresponding retinal thickness change over this interval is shown at right. (**b**) AO-OCT composite C-scan and B-scan images (*left*) indicating the imaged retinal regions, with magnified views of the examined locations (*right*) at baseline (*top*) and 2 years later (*bottom*). *Green* and *blue boxes* denote the retinal regions imaged by AO-OCT at baseline and at the 2-year follow-up, respectively. *Yellow dashed box* denotes the retinal location analyzed for soma loss. White *scale bars* denote 100 µm.

In the glaucoma subjects, we made fewer longitudinal measurements (T0 and T2) over a shorter follow-up period (2 years), and we found substantially fewer GCL somas (approximately 24× less) present within their arcuate defects compared with the six healthy subjects (see Table 3). These factors limited the accuracy of our loss rate estimates, but measurable soma loss was observed in both subjects. Figure 5 and Supplementary Figure S10 show representative AO-OCT images at the 2 timepoints and corresponding Spectralis GCL thickness maps for subjects G142 and G147, respectively.

Subject G142 exhibited a soma loss rate of −0.26%/yr, comparable to that observed in the healthy subjects, consistent with minimal disease progression at the imaged arcuate defect location during the measurement period. No significant change in GCL thickness was detected by AO-OCT at this location or by Spectralis OCT within the surrounding 3 × 3 degrees region. Similarly, no significant change in soma density was measured.

In contrast, subject G147 exhibited a soma loss rate of −31.6%/yr, more than 100-fold greater than that observed in the healthy subjects. Consistent with this rapid soma decline, GCL thickness at the corresponding location decreased by approximately 13%/yr as measured by AO-OCT. Similarly, Spectralis OCT showed an 11.8%/yr reduction in GCL thickness within the surrounding 3 × 3 degrees region. This loss is visually evident in the AO-OCT images, revealing localized GCL thinning on the superior (foveal) side of the arcuate defect and coincident with pronounced GCL soma loss. A detailed breakdown of GCL soma density and thickness measurements is provided in Supplementary Table S2.

### Repeatability of Soma Counting and Loss Detection

The SD of repeated measurements of soma density was 3.3% of the mean density (mean ± SD = 4614 ± 151 somas/mm^2^) across the 2 graders and 10 volumes. Assessment and correction of inter-grader disagreement by a third grader reduced this repeatability error to 1.1% of the mean.

In addition, we tested repeatability of soma loss detection. The same graders measured soma loss by comparing volumes of the same GCL soma patch acquired 1 year apart. Across the 4 volume pairs examined, the graders showed 100% agreement in identifying lost somas (0 to 2 losses per pair), thus resulting in the same soma loss rate of 0.43%. However, the small number of volumes limits our ability to evaluate the sensitivity of soma loss detection.

## Discussion

In this study, we used AO-OCT to longitudinally track thousands of individual GCL somas over multiple years in both healthy subjects and patients with glaucoma, enabling direct measurement of soma loss rates. Here, we summarize our key findings and place them in the context of existing histologic and clinical literature.

### Performance in Identifying Somas and Their Loss

Accurate counting of densely packed GCL somas arranged in a three-dimensional mosaic in AO-OCT images is challenging. Because ground-truth GC soma counts are not available for in vivo AO-OCT imaging, direct validation of our soma counts is not possible. We therefore assessed counting accuracy by evaluating the consistency of measurements across sessions.

Our initial strategy of reducing counting error focused on optimizing GCL soma sharpness during AO-OCT acquisition, using a customized graphic user interface for real-time three-dimensional visualization of GCL somas, and using trained agents for manual soma identification. Despite these measures, we observed significant differences in soma counts obtained from the same retinal patch across different years. When counts from each year were evaluated independently, the mean F1 score (similarity index) was only 0.88. Our soma density measurements alone were thus insufficiently sensitive to detect the soma loss rate.

To improve detection performance, we incorporated an image alternation technique (described in Data analysis), which provided two key advantages. First, it transformed the cognitively demanding task of identifying somas in a single image into the simpler task of detecting and classifying differences between paired images. Second, cross-year image comparisons allowed us to mitigate the effects of image noise and variable image quality on density estimates. Together, these improvements yielded near perfect agreement among images acquired at the same retinal location (mean F1 score = 0.998). The residual disagreement (approximately 0.2%) included actual soma losses (0.10%/year) as well as other temporal image changes that could not be definitively attributed to soma loss, including soma migration (see Supplementary Video S10).

Finally, we note the presence of temporally stable image features that we could not definitively identify as somas because they were contiguous with neighboring structures, such as nerve fiber bundles, inner plexiform layers, and blood vessels. Although such image features contributed to errors in soma density estimates (coefficient of variance = 3%), they did not substantially affect estimates of soma loss rate. This is because the total number of tracked somas (the denominator of the loss-rate calculation) greatly exceeds the number of somas lost (the numerator).

### Improved Performance in Measuring Soma Density

On a coarser level, the reliability of our soma identification is also supported by the strong agreement between our GC soma density estimates and published histologic data (see Fig. 2 left). In a prior AO-OCT study, we reported an apparent undercount of somas near the fovea (at 1.5–3 degrees and 3–4.5 degrees), where the GCL is thickest, relative to histology^38^; at the time, we were unable to resolve this discrepancy.

In the present study, we addressed this issue by implementing a through-focus imaging strategy that effectively extended the imaging depth-of-focus (DOF).^42^ Specifically, AO-OCT images were acquired and averaged with the system focus placed at multiple depths spanning the full GCL thickness. This approach ensured that somas throughout the GCL appeared sharply in at least a subset of images. In addition, repeated imaging over time provided multiple observations of the same somas, allowing improved reconciliation of errors arising from variations in image quality and grader performance.

### Ganglion Cell Loss Rate in Healthy Subjects

Among the 6 healthy subjects (age range = 24 to 50 years old), a very large fraction of GCL somas (approximately 99.7%) remained stable over the 3-year study period (see Figs. 1, 3, 4). Despite this, all 6 subjects exhibited measurable soma loss at each timepoint, with a mean soma loss rate across subjects of −0.10%/yr, with a 95% CI of −0.09 to −0.11%/yr and a 95% prediction interval of +0.02 to −0.22%/yr. Soma losses were rare, apparently random, and isolated events in these subjects. Notably, we found no statistically significant difference in soma loss rate among the six subjects (2-way ANOVA; F(5, 20) = 0.37, *P* = 0.86, ω² = 0.00), including subject H053 who was under medication for ocular hypertension. Consistent with this finding, excluding H053 from the analysis had essentially no effect on the loss rate: 0.100%/yr (with H053) versus 0.098%/yr (without H053).

In addition, among the six subjects, no significant differences in loss rate were observed across the retinal locations examined. Collectively, these findings are consistent with the idea that GCL soma loss in healthy subjects is an extremely slow, widespread process that is intrinsically part of normal aging.

### Comparison of Ganglion Cell Loss Rate in Healthy Subjects With Histological Studies

Table 4 compares our mean soma loss rate of −0.10%/yr with those reported in the histological and clinical literature. For each study, the table summarizes the measured parameter, study type, retinal location, number of subjects or eyes, health status, age (mean and range), loss rate, and associated *P* value. Additional details are provided in the Supplementary Notes, including our methods for deriving loss rates and *P* values when these were not reported in the publication. As shown in Table 4, our reported soma loss rate is broadly consistent with the relatively small soma and axon loss rates reported in the histological studies (soma loss rate = −0.19 to −0.57%/yr^12^^–^^15^^,^^57^ and axon loss rate = −0.07 to −0.4%/yr), although our estimate is lower than all but one study.

**Table 4.** Comparison of GCL Soma Loss Rate Measured in Histological, Clinical, and AO-OCT Studies

Study	Measurement	Study Type	Retinal Location	*N* *	Subject Health	Age Mean, Y	Age, Range, Y	Soma Loss Rate, %/Y	*P* Value
Gao and Hollyfield (1992)	Soma count	Ex vivo	0.96 mm from fovea	6	Healthy	3 in both second and sixth decades	17–62	−0.37	<0.05
Curcio and Drucker (1993)	Soma count	Ex vivo	<5 mm from fovea	9	Healthy	52.6	27–82	−0.19	0.43^†^
Blanks et al. (1996)	Soma count	Ex vivo	<1.5 mm from fovea	12	Healthy	79.7	60–98	−0.57	0.016
Harman et al. (2000)	Soma count	Ex vivo	<5 mm from fovea	12	Healthy	47.7	16–76	−0.37	0.28^†^
Balazsi et al. (1984)	Axon count	Ex vivo	Optic nerve	16	Healthy	40.5	3.5–82	−0.32	(0.02)
Mikelberg et al. (1989)	Axon count	Ex vivo	Optic nerve	12/16	Healthy	35.9	3.5–82	−0.37	0.13^†^
Repka and Quigley (1989)	Axon count	Ex vivo	Optic nerve	19	Healthy	55.8	4–84	−0.07	0.65^†^
Jonas et al. (1992)	Axon count	Ex vivo	Optic nerve	56 (72^‡^)	Healthy	54.7	19–88	−0.29	0.007
Morrison et al. (1990)	Axon count	Ex vivo	Optic nerve	28	Healthy (rhesus)	14.9	1.5–29	−0.4	0.07^†^
Fortune et al. (2014)	Axon count	Ex vivo	Optic nerve	83	Healthy (rhesus)	10	1.2–26.7	−0.11	0.74^†^
Harwerth and Wheat (2007)	24-2 VF	In vivo	<24 degrees from fovea	NA	Healthy	NA	25–95	−0.59	sig^∥^
Harwerth et al. (2008)	24-2 VF + cpRNFL	In vivo	<24 degrees from fovea + cpRNFL	55	Healthy	44.5	18–80	−0.46	<1e–4
Medeiros et al. (2012)	24-2 VF + cpRNFL	In vivo	<24 degrees from fovea + cpRNFL	52	Healthy	NA	NA	−0.7	<0.001
				47 (of 213)	Progressive glaucoma	60 ± 11 (213)	NA	−4.4	sig^∥^
Hirooka et al. (2016)	30-2 VF + cpRNFL	In vivo	<30 degrees from fovea + cpRNFL	41 (of 116)	Progressive glaucoma	61.7 ± 10.7 (116)	NA	−4.9	sig^∥^
Present study	Soma count	In vivo	<4 mm from fovea	6	Healthy	34.2^§^	24–50^§^	−0.10	<1e–3
			Arcuate defect	2	Glaucoma	NA	61–71	−0.26 or −31.65	NA

* Number of subjects in study.

† Soma loss rates are not significantly different from zero (*P* value > 0.05).

‡ Total number of eyes from the 56 subjects.

§ Age at first imaging session.

∥ Reported as statistically significant.

Interpreting this overall difference is difficult because of fundamental methodological differences between the studies, most notably the cross-sectional design of the histological studies compared with the longitudinal design of our study. Additional factors, including differences in subject age, retinal sampling location, and the relatively small sample sizes, may also have contributed to the observed discrepancies. Notably, all of the histological studies examined older eyes, on average, than those included in our study. In particular Blanks et al. studied only subjects aged 60 to 98 years, substantially older than our cohort, and reported the largest estimated GCL soma loss rate (−0.57%/yr).

It is also noteworthy that only 3 of the 8 histological studies in Table 4 reported GCL soma or axon loss rates that were significantly different from zero (*P* < 0.05): Gao and Hollyfield,^12^ Blanks et al.,^14^ and Jonas et al.^18^ However, when Jonas et al. restricted their analysis to subjects younger than 60 years (*N* = 26)—an age range more comparable to our cohort—the estimated loss rate was no longer statistically significant.

Taken together, these findings indicate that there is limited histological evidence for age-related GCL soma or axon loss in healthy subjects younger than 60 years, making direct comparison with our longitudinal estimate speculative. Nevertheless, the lower GCL soma loss rate observed in our study compared with the histological estimates is consistent with the possibility that GC loss accelerates with age. Additional longitudinal studies spanning a wider age range will be required to test this hypothesis.

### Comparison of Ganglion Cell Loss Rate in Healthy Subjects to Clinical Studies

We know of no prior study that has directly measured the rate of GCL soma loss in either healthy subjects or patients with diseased eyes using AO-OCT or any other cellular-level imaging modality. Thus, there is no directly comparable in vivo study against which to evaluate our findings. Furthermore, comparisons with extensive literature based on visual field testing and retinal layer thickness measurements are inherently challenging because these methods assess fundamentally different structural and functional metrics. That said, some qualitative comparisons may provide useful context for interpreting our results.

As shown in Table 4, clinical studies with visual field testing have reported rates of GC loss from −0.46 to −0.7%/yr.^26^^,^^28^^,^^58^ Harwerth et al. estimated a loss rate of −0.59%/yr using a model relating visual field sensitivity to neuronal loss.^26^ In a subsequent refinement, the same group combined visual field sensitivity measurements with 360-degree cpRNFLT measurements, yielding an estimated GC soma loss rate of −0.46%/yr.^28^ Similarly, Medeiros et al. integrated 24-2 visual field sensitivity measurements with cpRNFLT measurements and reported a GC loss rate of −0.70%/yr.^58^ These estimates are five to seven times higher than the mean soma loss rate observed in our study. A loss rate of −0.5 to −0.7%/yr would correspond to the disappearance of 12 to 17 somas within each of our retinal patches over a 3-year period, changes that would have been readily detectable in our AO-OCT images.

Clinical studies using only OCT examined the effects of aging on cpRNFL and macular inner retinal layer thicknesses. Increasing evidence supports a statistically significant effect of age on cpRNFLT,^59^^–^^71^ macular GC complex (NFL + GCL + IPL) thickness,^70^^–^^72^ macular GCIPL (GCL + IPL) thickness,^67^^,^^70^^,^^73^ and macular GCL thickness.^68^^,^^74^ Reported NFL and GCL thinning rates of approximately −0.2 ± 0.1%/yr are similar across studies.^59^^–^^74^ Notably, our mean soma loss rate of −0.10%/yr is reasonably close to the reported thinning rates in these large-scale clinical OCT studies (approximately −0.2%/yr). This correspondence is perhaps not surprising given the strong correlation we measured between GCL thickness and soma density in the six healthy subjects (*R^2^* = 0.96, *P* < 0.001).

Overall, comparisons with previous clinical studies reveal substantial variability in reported rates of age-related loss. Estimates derived from retinal layer thickness measurements are broadly consistent with our AO-OCT measurements, whereas those inferred from combined visual field sensitivity and RNFLT suggest faster rates of loss. The reasons for these discrepancies remain unclear and likely reflect a combination of methodological differences (including small sample sizes), biological variability (including differences in ages), and assumptions underlying the models used to infer neuronal loss. Further studies are needed to determine how direct AO-OCT measurements of GCL soma loss relate to these established clinical metrics and to establish the role of cellular-resolution imaging within the broader framework of age-related retinal change.

### Ganglion Cell Loss Rate in Glaucoma Subjects

We measured GC loss in two glaucoma subjects. The loss rate in G142 (−0.26%/yr) was somewhat higher than the mean loss rate observed in the healthy subjects (−0.10%/yr). Because this subject had a relatively low baseline soma density, a rate of −0.26%/yr corresponds to the loss of only a single soma over the 2.3-year study period. Nevertheless, this loss exceeds the prediction interval (+0.02 to –0.22%/yr) associated with our mean loss rate of −0.10%/yr. This comparison assumes that the loss rate measured in our healthy cohort also applies to older individuals comparable in age to G142, an assumption that remains unverified. GCL thickness, measured by both AO-OCT and Spectralis OCT at the same arcuate defect location, showed no detectable thinning over the study period (see Fig. 5). Collectively, these findings are consistent with minimal disease progression in the sampled defect region.

By contrast, the GC loss rate in G147 (−31.65%/yr) was more than 2 orders of magnitude greater than the mean loss rate of the healthy subjects, again assuming this loss rate applies to older individuals. GCL thickness maps demonstrate localized thinning at the AO-OCT imaged location (see Supplementary Fig. S10). GCL thickness measurements at the arcuate defect location declined by −13%/yr (AO-OCT) and −17.7%/yr (Spectralis OCT), indicative of focal glaucomatous damage and likely explaining the markedly elevated loss rate. Together these results indicate rapid local disease progression within the arcuate defect region.

Note that both glaucoma subjects received IOP-lowering treatment throughout the study. Because IOP-lowering treatment is known to slow glaucoma progression, the measured GC loss rates likely underestimate those that would have occurred without treatment. However, the present study was not designed to evaluate the effect of treatment versus no treatment or to compare the effects of different IOP-lowering or neuroprotective treatments.

Our findings can be compared with previous clinical studies that estimated GC loss rates from conventional structural and functional measurements. Medeiros et al. reported progression in 47 of 213 eyes with glaucoma, with a mean loss rate of −4.4%/yr (range = −1.1 to −10.5%/yr).^58^ A similar, more recent structure-function study by Hirooka et al.^75^ reported a similar mean loss rate of −4.9%/yr, detecting progression in 41 of 116 glaucomatous eyes. These studies are summarized in Table 4.

The loss rates measured in our two glaucoma subjects span a substantially wider range than those reported in these clinical studies. G142 exhibited a loss rate (−0.26%/yr) that was more than an order of magnitude lower than the reported clinical averages, whereas G147 exhibited a loss rate (−31.6%/yr) approximately 7 times greater. Comparison beyond this, however, is challenging because our measurements were obtained from localized AO-OCT imaging of specific retinal regions, whereas the clinical studies estimated loss rates from measurements averaged over much larger retinal areas. In addition, glaucoma progression is highly heterogeneous, varying both between retinal locations within an eye and between subjects.

Interestingly, the reported loss rates in the clinical studies were limited to those eyes in which progression was detectable, representing 22%^58^ and 35%^75^ of their glaucoma cohorts. Given the sensitivity of AO-OCT to detect extremely small loss rates, including those associated with normal aging (−0.10%/yr; prediction interval = +0.02 to −0.22%/yr), any loss exceeding −0.22%/yr would meet our criterion for progression. With this level of sensitivity, we would expect progression to be detectable in a substantially larger fraction of glaucoma subjects than the 22% to 35% reported in these clinical studies.

### Relationship Between GC Soma Density and GCL Thickness Measured With AO-OCT

As shown in Figure 2, we observed a strong linear correlation between GC soma density and GCL thickness in the healthy subjects (GC density = 645 × GCL thickness − 6628, *R^2^* = 0.96, *P* < 0.001). This linear relationship is similar to an earlier AO-OCT report^46^ but it is not necessarily expected—and may even be surprising—because both GC soma size and distribution of soma sizes vary markedly with retinal eccentricity,^38^ as does GCL thickness. Moreover, although our GC soma density estimates were corrected by subtracting the proportion of displaced amacrine cells reported by Curcio and Allen (1990),^1^ the strong linear relationship was preserved even when the amacrine cells were included.

This linear relationship was not preserved in the arcuate defect of subject G147, where the GCL soma loss rate (−31.65%/yr) was approximately 2.4× greater than the GCL thinning rate measured at the same location with AO-OCT (−13%/yr). Although this apparent dissociation could be the result of soma loss outpacing GCL thinning as disease progresses, we suspect that increased local variability in GCL thickness in advanced disease substantially degrades the reliability of thickness as a surrogate for soma density.

Specifically, we find that thickness variability increases as the GCL thins due to the relative prominence of blood vessels within the layer, the formation of GCL regions (or pockets) devoid of somas, the tendency of surviving somas to cluster around blood vessels,^47^^,^^51^ and increased spacing between NF bundles containing a mixture of somas, Mueller cell processes, and extracellular components. We also find surviving somas in regions where the local average GCL thickness is thinner than the residual somas that occupy it (approximately 12 µm). Collectively, these factors support the idea that GCL thickness becomes a progressively less reliable predictor of GCL soma density as glaucoma advances.

### Limitations of the Study

Key limitations of this study include the small number of subjects, retinal locations, and longitudinal timepoints examined. Consequently, the study was not sufficiently powered to derive population-level estimates of GCL soma loss or to evaluate the effects of age, sex, and other biological variables on the rate of GCL soma loss. In addition, further improvements in AO-OCT resolution would enhance visualization of the GCL somas, particularly in older subjects and in eyes with disease. Another limitation is that only the first step of the multi-step, multi-grader procedure for determining GCL soma loss was masked to the grader (i.e. identifying the total number of GCL somas present). The second step, which involved reconciling and correcting soma counts across images, was not masked. Consequently, this step may have introduced selection bias, although it substantially improved the identification of soma loss between imaging sessions.

It is unlikely that microglial cells—which comprise a small fraction of retinal cells and reside primarily in the inner and outer plexiform layers—contributed to the soma losses observed in the GCL. Although we have not yet identified microglia in our AO-OCT images, homeostatic microglia exhibit a characteristic ramified morphology and highly dynamic process motility with limited cell-body displacement over days to weeks. In contrast, the GCL soma mosaic remained remarkably stable throughout the multi-year imaging period, with soma loss occurring only as rare, isolated events. If some of the losses instead reflected microglial displacement or movement rather than true GC loss, we would expect to observe a comparable number of newly appearing cells (i.e. false positives) elsewhere in the mosaic. We observed no such events or ramified cells, providing no evidence that microglial cells contributed to our measurements of GC loss.

Finally, manual identification of GCL somas is labor-intensive and time-consuming, which fundamentally constrains scalability and clinical translation. In an attempt to address this latter challenge, we applied machine-learning software^46^ for soma identification. Although the software's accuracy was comparable to, or in some cases exceeded, that of manual counting at a single timepoint, it was insufficient to reliably detect the small percentage changes in soma density associated with longitudinal GCL soma loss. Thus, we were unable to use it for this purpose; further development will be needed to overcome these limitations.

## Conclusions

We present a novel AO-OCT–based method for longitudinally tracking individual GCL somas and quantifying their loss in both healthy subjects and patients with glaucoma. In healthy subjects, the GCL soma mosaic remained highly stable over several years, with cell loss occurring as rare, isolated events. By contrast, glaucoma subjects exhibited higher annual soma loss rates and higher inter-subject variability. These findings establish that direct in vivo measurement of individual GC soma loss is now feasible and may offer a powerful tool for longitudinal monitoring of glaucoma suspects and for assessing the efficacy of therapeutic interventions.

## Supplementary Material

Supplement 1

Supplement 2

Supplement 3

Supplement 4

Supplement 5

Supplement 6

Supplement 7

Supplement 8

Supplement 9

Supplement 10

Supplement 11

## Supplementary Material

Supplementary Video S1. Animation demonstrating the use of the customized graphic user interface to identify and mark GCL somas. The interface displays simultaneous en face (XY) and cross sectional (XZ and YZ B-scan) views of an example AO-OCT volume. The grader analyzes the entire GCL volume, identifying each soma and its 3D coordinates based on its three-dimensional appearance. This process is facilitated by moving the 3D yellow crosshairs (cursor position) in real time to the volumetric center of each soma. Each identified soma is marked with a red ‘x.’ The soma's 3D size is then measured, and a 3D oval of the same size is added to the AO-OCT volume. Each oval is assigned a random color to distinguish adjacent somas. Color ovals are then superimposed onto the interface displays, which helps confirm that (1) their center coordinates and size estimation are reasonable and (2) there are no missing or overlapping somas.

Supplementary Video S2. Animation demonstrating the image alternation technique to facilitate detection of soma loss. The technique was incorporated into the customized graphic user interface. Each soma was independently assessed using the alternation method with the depth position optimized for that soma. Somas identified at baseline and at the 1-year follow-up are marked with red and green ‘x’ symbols, respectively. For a visualization purpose, these symbols are superimposed onto the interface display after projecting them along the depth. Displaying a difference between two coordinates facilitates the process for detecting soma loss while correcting errors.

Supplementary Video S3. Time-lapse view of GCL somas imaged in subject H053 at seven retinal locations across four timepoints. The top row shows AO-OCT B-scan images, and the bottom row shows corresponding en face images at baseline and at the 1-, 2-, and 3-year follow-up visits.

Supplementary Video S4. Time-lapse view of GCL somas imaged in subject H116 at five retinal locations across four timepoints. The top row shows AO-OCT B-scan images, and the bottom row shows corresponding en face images at baseline and at the 1-, 2-, and 3-year follow-up visits.

Supplementary Video S5. Time-lapse view of GCL somas imaged in subject H125 at five retinal locations across four timepoints. The top row shows AO-OCT B-scan images, and the bottom row shows corresponding en face images at baseline and at the 1-, 2-, and 3-year follow-up visits.

Supplementary Video S6. Time-lapse view of GCL somas imaged in subject H127 at five retinal locations across four timepoints. The top row shows AO-OCT B-scan images, and the bottom row shows corresponding en face images at baseline and at the 1-, 2-, and 3-year follow-up visits.

Supplementary Video S7. Time-lapse view of GCL somas imaged in subject H154 at five retinal locations across two timepoints. The top row shows AO-OCT B-scan images, and the bottom row shows corresponding en face images at baseline and at the 1-year follow-up visit.

Supplementary Video S8. Time-lapse view of GCL somas imaged in subject H155 at five retinal locations across two timepoints. The top row shows AO-OCT B-scan images, and the bottom row shows corresponding en face images at baseline and at the 1-year follow-up visit.

Supplementary Video S9. Example of a GCL soma loss event at 1.5–3° retinal eccentricity in subject H053. The loss occurs at the yellow cursor position between baseline and the 1-year follow-up visit.

Supplementary Video S10. Example of a GCL soma migration event at 3–4.5° retinal eccentricity in subject H053. The upward (anterior) migration occurs at the yellow cursor position between the 2- and 3-year follow-up visits.
